# Stemness Refines the Classification of Colorectal Cancer With Stratified Prognosis, Multi-Omics Landscape, Potential Mechanisms, and Treatment Options

**DOI:** 10.3389/fimmu.2022.828330

**Published:** 2022-01-27

**Authors:** Zaoqu Liu, Hui Xu, Siyuan Weng, Yuqing Ren, Xinwei Han

**Affiliations:** ^1^ Department of Interventional Radiology, The First Affiliated Hospital of Zhengzhou University, Zhengzhou, China; ^2^ Interventional Institute of Zhengzhou University, Zhengzhou, China; ^3^ Interventional Treatment and Clinical Research Center of Henan Province, Zhengzhou, China; ^4^ Department of Respiratory and Critical Care Medicine, The First Affiliated Hospital of Zhengzhou University, Zhengzhou, China

**Keywords:** stemness, colorectal cancer, multi-omics, machine learning, immunity

## Abstract

**Background:**

Stemness refers to the capacities of self-renewal and repopulation, which contributes to the progression, relapse, and drug resistance of colorectal cancer (CRC). Mounting evidence has established the links between cancer stemness and intratumoral heterogeneity across cancer. Currently, the intertumoral heterogeneity of cancer stemness remains elusive in CRC.

**Methods:**

This study enrolled four CRC datasets, two immunotherapy datasets, and a clinical in-house cohort. Non-negative matrix factorization (NMF) was performed to decipher the heterogeneity of cancer stemness. Multiple machine learning algorithms were applied to develop a nine-gene stemness cluster predictor. The clinical outcomes, multi-omics landscape, potential mechanisms, and immune features of the stemness clusters were further explored.

**Results:**

Based on 26 published stemness signatures derived by alternative approaches, we decipher two heterogeneous clusters, low stemness cluster 1 (C1) and high stemness cluster 2 (C2). C2 possessed a higher proportion of advanced tumors and displayed worse overall survival and relapse-free survival compared with C1. The MSI-H and CMS1 tumors tended to enrich in C1, and the mesenchymal subtype CMS4 was the prevalent subtype of C2. Subsequently, we developed a nine-gene stemness cluster predictor, which robustly validated and reproduced our stemness clusters in three independent datasets and an in-house cohort. C1 also displayed a generally superior mutational burden, and C2 possessed a higher burden of copy number deletion. Further investigations suggested that C1 enriched numerous proliferation-related biological processes and abundant immune infiltration, while C2 was significantly associated with mesenchyme development and differentiation. Given results derived from three algorithms and two immunotherapeutic cohorts, we observed C1 could benefit more from immunotherapy. For patients with C2, we constructed a ridge regression model and further identified nine latent therapeutic agents, which might improve their clinical outcomes.

**Conclusions:**

This study proposed two stemness clusters with stratified prognosis, multi-omics landscape, potential mechanisms, and treatment options. Current work not only provided new insights into the heterogeneity of cancer stemness, but also shed light on optimizing decision-making in immunotherapy and chemotherapy.

## Introduction

In 2020, approximately 1,880,000 new cases worldwide are diagnosed with colorectal cancer (CRC), resulting in around 910,000 deaths (1). Although the clinical outcomes of CRC patients have improved with the development and diversification of cancer treatment, tumor relapse and metastasis remain the leading causes of death for CRC (2). Recently, the high heterogeneity and molecular complexity of CRC have become evident (2–4). For example, patients with *KRAS* mutation are resistant to cetuximab and have a strong potential for tumor relapse (5). *TTN* and *OBSCN* commutation suggests favorable prognosis and abundant immune infiltration in CRC (6). Based on eight extracted mutational signatures, our team has previously proposed two heterogenous subtypes with distinct clinical outcomes and molecular alterations (7). Overall, the diverse clinical outcomes may be directly or indirectly mirrored at the molecular and cellular level of the heterogenous tumor microenvironment. Over the past decade, the tremendous advancement in next-generation sequencing has brought deciphering codes for tumor heterogeneity (3, 4, 8), and it is promising to generate new insights and develop more effective tools for clinical management.

Elegant studies have indicated that a few tumor cells could present stem cell-like features, termed cancer stem cells (CSCs) (9). Stemness refers to the capacities of self-renewal and repopulation, which contributes to tumor progression, relapse, and drug resistance (10, 11). As is well known, 5-Fluorouracil based chemotherapy is the first line option for CRC, which could eliminate the residual cancer cells after surgery for some patients (12). Of note, relapse after chemotherapy remains the obstacle to improving clinical outcomes of CRC patients, a critical mechanism is that 5-Fluorouracil elevates CRC stemness by activating TP53-mediated WNT signaling pathway (13). Previous studies also reported that some molecules could overcome or promote stemness (5, 14–17), which could further lead to shifts in biological behavior and heterogeneity of clinical outcomes.

Mounting evidence have established the links between cancer stemness and intratumoral heterogeneity across cancer (2, 9, 10, 18), and a theory believes cancer stemness is the origin of intratumoral heterogeneity and plasticity (19). Currently, the intertumoral heterogeneity of cancer stemness remains elusive in CRC. A pan-cancer study has established a one-class logistic regression model (OCLRM) to measure two stemness indices from transcriptomic and epigenetic data in bulk samples (11). However, the prediction of OCLRM is not reliable in CRC, as cancer stemness increases, patients have better prognosis and tumor stage, which violates common sense and might derive from the overfitting of supervised machine learning model (20).

This study aimed to investigate the associations between intertumoral heterogeneity and cancer stemness. Based on 26 published stemness signatures derived by alternative approaches, unsupervised clustering was performed to identify heterogeneous stemness clusters in clinical samples. We further characterized the clinical outcomes, multi-omics landscape, potential mechanisms, and immune features of the stemness clusters. The stemness clusters also exhibited different response rates to immunotherapy. In addition, using large-scale drug screening and molecular data, nine potential agents were identified for high stemness cluster to improve current therapeutic strategies in CRC.

## Material and Method

### RNA-Sequencing Cohort

The Cancer Genome Atlas Colon Adenocarcinoma and Rectal Adenocarcinoma (TCGA-CRC) included 616 eligible CRC patients who underwent primary curative resection. Transcriptome profiling data (HTSeq-Counts), somatic mutation data (VarScan2), copy number variation (CNV) data (Masked Copy Number Segment), and clinical data were retrieved from the TCGA portal (https://portal.gdc.cancer.gov). The raw count expression was transformed into transcripts per kilobase million (TPM).

### Microarray Cohorts

Three CRC microarrays (based on the GPL570 platform) were collected from the GEO database, including GSE39582 (n =585), GSE87211 (n =363), and GSE39084 (n =70). The raw expression data were processed and normalized using the robust multiarray average (RMA) algorithm implemented in the *Affy* package. The survival data were generated from the series matrix files.

### In-House Cohort

A total of 72 CRC patients who underwent primary curative resection at the First Affiliated Hospital (Zhengzhou University, Henan, China) were enrolled. All patients gave written informed consent, and none of the patients received any preoperative chemotherapy or radiotherapy. The detailed baseline was illustrated in **Table S1**. This project was approved by the Ethics Committee Board of The First Affiliated Hospital of Zhengzhou University. Quantitative real-time PCR (qRT-PCR) was performed to quantify the expression level of several key genes. The primer sequences were shown in **Table S2**. See **Supplementary Material** for details.

### Immunotherapy Cohorts

Two cohorts (including GSE35640 and IMvigor 210) with both expression data and immunotherapeutic information were also enrolled. The GSE35640 includes 56 patients with metastatic melanoma based on the GPL570 platform and the IMvigor 210 dataset includes 298 patients with metastatic urothelial cancer based on the Illumina sequencing platform. The raw expression data of GSE35640 were normalized by RMA approach and the IMvigor 210 dataset was processed *via* the *IMvigor210CoreBiologies* package. The therapeutic benefit was assessed *via* the RECIST v1.1 standard, complete response (CR)/partial response (PR) and stable disease (SD)/progressive disease (PD) were considered as responder and non-responder, respectively.

### Cancer Cell Line Data

The RNA sequencing expression data (TPM normalized) for 1019 cell lines were achieved from the Cancer Cell Line Encyclopedia (https://sites.broadinstitute.org/ccle). Drug sensitivity information of cell lines was obtained from two pharmacogenomic datasets, CTRP and PRISM, which provide large-scale drug screening and molecular data across hundreds of cancer cell lines. The area under the dose-response curve (AUC) values is a measure of drug sensitivity. For a specific drug, the lower the AUC value, the higher the sensitivity. Cell lines derived from hematopoietic and lymphoid tissues were removed. Drugs with more than 80% of non-missing data were retained, and then K-nearest neighbor using a Euclidean metric was applied for missing data imputation.

### Stemness Signatures

The StemChecker webserver (http://stemchecker.sysbiolab.eu) curated 26 published stemness signatures (Homo sapiens) derived by alternative approaches, such as expression profiles, computationally derived, literature curation, transcription factor target genes, and RNAi screen methods (21). In the TCGA-CRC dataset, we employed gene set variation analysis (GSVA) to measure the enriched abundance of each sample across the 26 stemness signatures.

### Non-Negative Matrix Factorization

To identify heterogeneous stemness subtypes in CRC, we performed non-negative matrix factorization (NMF) on the stemness score data to decipher the enrichment pattern of stemness signatures (22). Because the NMF algorithm prohibits negative elements within the matrix, we further normalize data with the min-max scaling, which could rescale the range of features to scale the range in [0, 1]. In the NMF framework implemented in the *NMF* package, we used the number of runs to perform = 100, method = “lee”, and potential factorization ranks = 2-9. To determine the optimal rank, the cophenetic coefficient and silhouette statistic were utilized to evaluate the stability of factorization. The first rank for which the cophenetic coefficient starts decreasing was generally defined as the optimal rank (22). The silhouette statistic quantifies how similar a sample is to its own cluster relative to other clusters, a high silhouette indicates that the sample is well matched to its own cluster and poorly matched to neighboring clusters (23). The principal component analysis (PCA) algorithm further assessed the robustness of clusters according to the two-dimension spatial distribution.

### Multiple Machine Learning Algorithms Derived the Stemness Cluster Predictor

The 616 patients from the TCGA-CRC dataset were randomly divided into training (70%, n =432) and testing (30%, n =184) datasets using *createDataPartition* function implemented in the *caret* package. Subsequently, the stemness cluster predictor was developed according to the following pipeline:

(1) The receiver operating characteristic (ROC) curves were utilized to select genes with the area under the ROC curve (AUC) greater than 0.65 for predicting stemness clusters.
(2) In the training dataset, four machine learning algorithms, including least absolute shrinkage and selection operator (LASSO) regression, support vector machine (SVM), random forest (RF), and extreme gradient boosting (XGBoost), were conducted to further identify the key genes with important information for the stemness clusters.
(3) Furthermore, the overlap genes determined by the four algorithms were subject to the logistic regression analysis for establishing the stemness cluster predictor.
(4) The performance of our stemness cluster predictor was validated in the internal testing dataset, three external validation datasets (including GSE39582, GSE87211, and GSE39084), and an in-house dataset (qRT-PCR).

### Multi-Omics Landscape of the Stemness Clusters

To delineate the multi-omics landscape of the stemness clusters, we integratively analyzed the mutation and CNV data in the TCGA-CRC dataset. According to the previous studies (6, 24), genes with the top 20 mutational frequency were defined as the frequently mutated genes (FMGs), and genes with the top 15 amplification (AMP) or homozygous deletion (Homdel) were defined as the frequently AMP or Homdel genes (FAGs/FHGs). The tumor mutation burden (TMB), single-nucleotide polymorphism (SNP), and insertion and deletion (Indel) were defined as the numbers of total mutations, single-nucleotide mutations, insertion and/or deletion of nucleotides into the DNA sequence, respectively. To quantify the genomic alteration in the stemness clusters, we calculated the fraction of genome alteration (FGA), fraction of genome gained (FGG), and fraction of genome lost (FGL), defined as the ratio of total CNV/gain/lost bases to all bases, respectively. In addition, based on the recurrently altered regions derived from the GISTIC 2.0 pipeline, the burdens with copy number changes at the focal and arm levels were quantified.

### Gene Set Enrichment Analysis

Gene set enrichment analysis (GSEA) is a bioinformatics algorithm that transforms information from gene-level into pathway-level. A total of 21338 gene sets included gene ontology (GO), Kyoto encyclopedia of genes and genomes (KEGG), and hallmark information from the Molecular Signatures Database (MSigDB) resource (version 7.4, h.all.v7.4.symbols.gmt, c2.all.v7.4.entrez.gmt, and c5.all.v7.4.entrez.gmt). All genes were sorted by the descending log_2_-transformation fold change (log_2_FC) between the two stemness clusters, and then the *clusterProfiler* package was performed for GSEA analysis. Gene terms with false discovery rate (FDR) <0.001 were enriched in the stemness clusters.

### Tumor Microenvironment Profiles

The Microenvironment Cell Populations-counter (MCP-counter) approach was utilized to quantify the absolute infiltration abundance of eight immune and two stromal cell subpopulations from the transcriptomic profiles of TCGA-CRC (25). The expression patterns of 27 immune checkpoint molecules between the two stemness clusters were further explored (26–29).

### Assessment of Immunotherapeutic Efficacy

According to the expression profiles of each sample from the TCGA-CRC dataset, we evaluated the immunotherapeutic efficacy between the two stemness clusters *via* three distinct algorithms, including Tumor Immune Dysfunction and Exclusion (TIDE), T-cell-inflamed gene-expression profile (GEP), and subclass mapping (SubMap). A high TIDE score suggests a worse immunotherapeutic efficacy, while a high T-cell-inflamed GEP indicates an increasing tendency to benefit from PD-1 blockade (30, 31). The SubMap was performed to measure the expression similarity between the two stemness clusters and immunotherapeutic groups with different responses (32).

### Potential Therapeutic Agents

Two pharmacogenomic datasets, CTRP and PRISM, provide large-scale drug screening and transcriptomic profiles across hundreds of cancer cell lines, which makes it possible to precisely predict drug response in clinical tissues (33). For drug response prediction, the ridge regression model implemented in the *pRRophetic* package was performed to assess drug response of clinical tissues (34). The predictive model was trained on transcriptomic profiles and drug sensitivity data of cancer cell lines using the 10-fold cross-validation, allowing the estimation of drug response in the TCGA-CRC dataset. Subsequently, differential analysis between the stemness clusters for drug sensitivity was performed to identify potential therapeutic agents.

### Statistical Analysis

All data processing, visualization, and statistical analysis were performed in the R 4.0.5 software. Kolmogorov-Smirnov test detected data normality. T-test or Wilcox-test were performed to compare quantitative variables, while Chi-square test was used to compare qualitative variables. Kaplan-Meier survival analysis was performed *via* the *survival* package. The consensus molecular subtype (CMS) information of each sample was obtained from the *CMSclassifier* package. Based on the previous published six classification systems, Guinney et al. proposed four CMS subtypes with distinguishing traits, for example, CMS1 was characterized with hypermutated, microsatellite unstable and strong immune activation (35). The *pROC* package was applied to plot the ROC curves. A two-sided *P <*0.05 was considered statistically significant. Microsatellite instability-high (MSI-H) CRC refers to tumors with a high degree instability due to the dysfunction of mismatch repair genes.

## Results

### Identification of Two Robust Stemness Clusters in CRC

**Figure 1:**
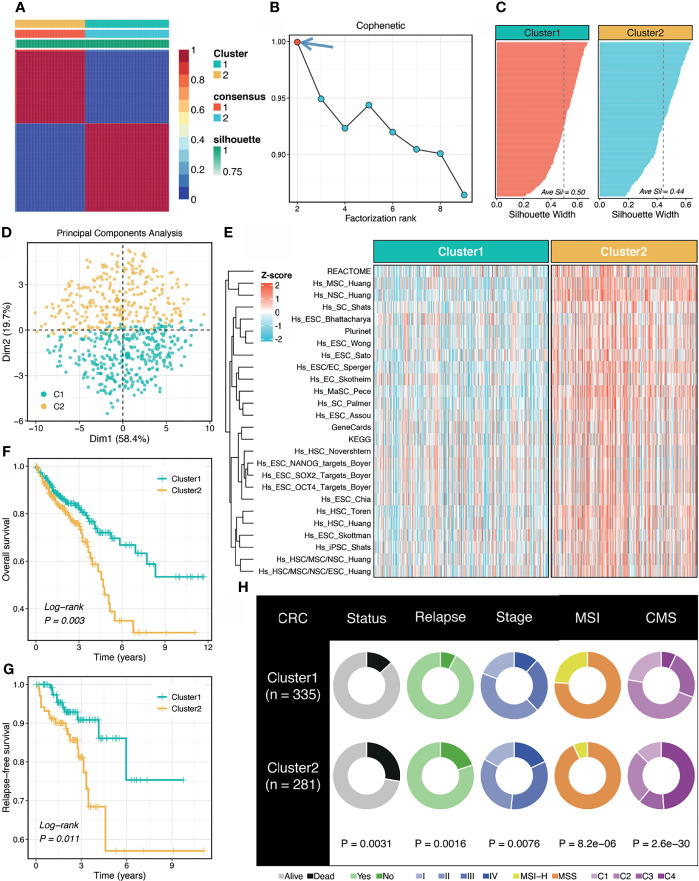
Non-negative matrix factorization (NMF) identified two stemness clusters with heterogeneous prognosis and clinical features. **(A)** The consensus map of NMF clustering results in the TCGA-CRC dataset. **(B)** The first rank (= 2) for which the cophenetic coefficient starts decreasing was generally defined as the optimal rank. **(C)** The silhouette statistic of two stemness clusters. **(D)** The principal component analysis (PCA) algorithm displayed the two-dimension spatial distribution of two clusters. **(E)** Heatmap demonstrated C2 possessed significantly higher stemness abundance compared with C1. **(F, G)** Kaplan-Meier curves of OS **(F)** and RFS **(G)** according to the stemness clusters. **(H)** Distributions of clinical features between two clusters.

According to the abundance of 26 published stemness signatures calculated using the GSVA framework, the NMF approach was performed to detect the factorization rank. The consensus matrices demonstrated stable and excellent discrimination at rank = 2 (**Figure 1A**). The cophenetic coefficient started decreasing at rank = 2, which indicated the optimal rank is 2 (**Figure 1B**). Samples within two clusters were also well matched to their own cluster compared with another cluster according to the high silhouette statistics (**Figure 1C**). In addition, PCA further displayed the separable two-dimension spatial distribution of two clusters (**Figure 1D**). Thus, we eventually identified two robust stemness clusters. As shown in **Figure 1E**, cluster 2 (C2) possessed significantly higher stemness abundance compared with cluster 1 (C1), which suggested an enhanced cancer stemness and more poor-differentiated tumors in C2.

### Stemness Cluster 2 Had Worse Prognosis and Clinical Features

The Kaplan-Meier survival analysis suggested C2 displayed unfavorable overall survival (OS) and relapse-free survival (RFS) (*P <*0.05, **Figures 1F, G**). The mortality and relapse rate in C2 was dramatically higher than C1 (*P <*0.05, **Figure 1H**). The tumor stage of C2 tended to be progressive (*P <*0.05, **Figure 1H**). In addition, C1 possessed a higher proportion of MSI-H tumors, which was reported to have better clinical outcomes and higher infiltrating lymphocytes (*P <*0.05, **Figure 1H**). Based on the CMS information inferred from the *CMSclassifier* package, we observed that a higher proportion of CMS1 in C1 and of CMS4 in C2 (*P <*0.05, **Figure 1H**).

### Construction and Validation of a Stemness Cluster Predictor

**Figure 2:**
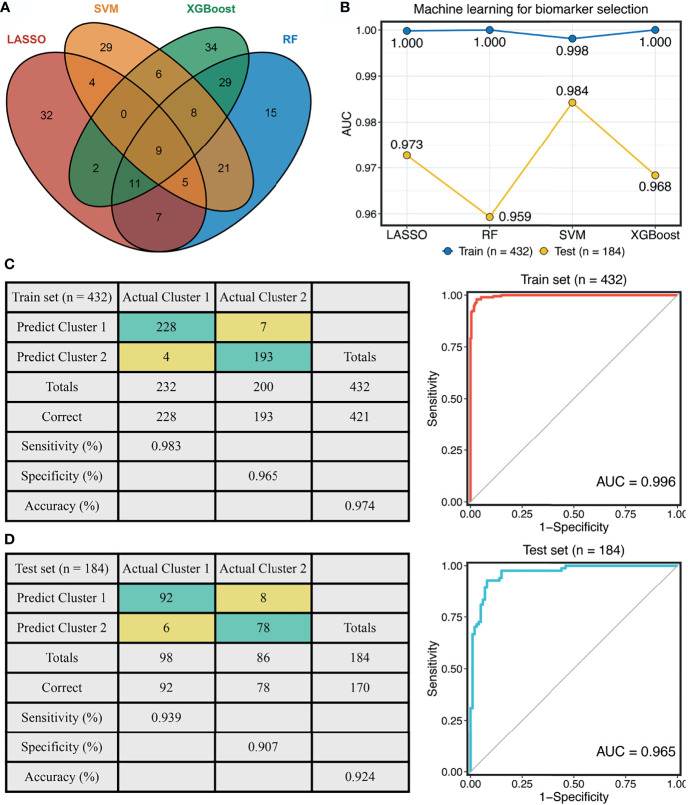
Construction of the stemness cluster predictor. **(A)** Venn diagram identified nine key stemness subtype specific genes that were shared by four feature selection algorithms. **(B)** The performances of four machine-learning algorithms for feature selection were evaluated in the training and testing sets. **(C, D)** The confusion matrices and ROC curves of the stemness clusters predictor in the training **(C)** and testing **(D)** sets.

To reproduce our subtype in other cohorts, we tried to construct a stemness cluster predictor. First, four machine learning algorithms were performed with 247 genes with AUC >0.65, to identify the key genes with important information for the stemness clusters. A total of 70, 82, 99, and 105 were determined *via* LASSO, SVM, XGBoost, and RF, respectively (**Figure 2A**). The ROC analyses exhibited that the four algorithms possessed excellent performance in feature selection, with AUCs of >0.99 and >0.95 for the TCGA training and testing sets, respectively (**Figure 2B**). In total, nine key genes were shared by the four algorithms. As shown in **Figure S1**, GFPT1, PTMAP9, MOGAT3, and DPM3 enriched in C1, while S100A12, PGM5, FUT6, SEMA3C, and ADAM33 overexpressed in C2. These suggested GFPT1, PTMAP9, MOGAT3, and DPM3 might indicate a lower stemness, while S100A12, PGM5, FUT6, SEMA3C, and ADAM33 were associated with a higher stemness in CRC. In addition, we further assessed the prognostic value of these nine genes. In line with the stemness predictions, GFPT1, PTMAP9, MOGAT3, and DPM3 prolonged OS, while S100A12, PGM5, FUT6, SEMA3C, and ADAM33 suggested a dismal OS (**Figure S2**). Based on the expression profiles of the nine genes, we fitted a logistic regression model for predicting the stemness clusters. Further analyses demonstrated that the stemness cluster predictor could precisely distinguish C1 and C2, with a sensitivity of 0.983, a specificity of 0.965, an accuracy of 0.974, and an AUC of 0.996 in the training set (**Figure 2C**). Likewise, the predictor also displayed an excellent performance in the testing set, with a sensitivity of 0.939, a specificity of 0.907, an accuracy of 0.924, and an AUC of 0.965 (**Figure 2D**).

**Figure 3:**
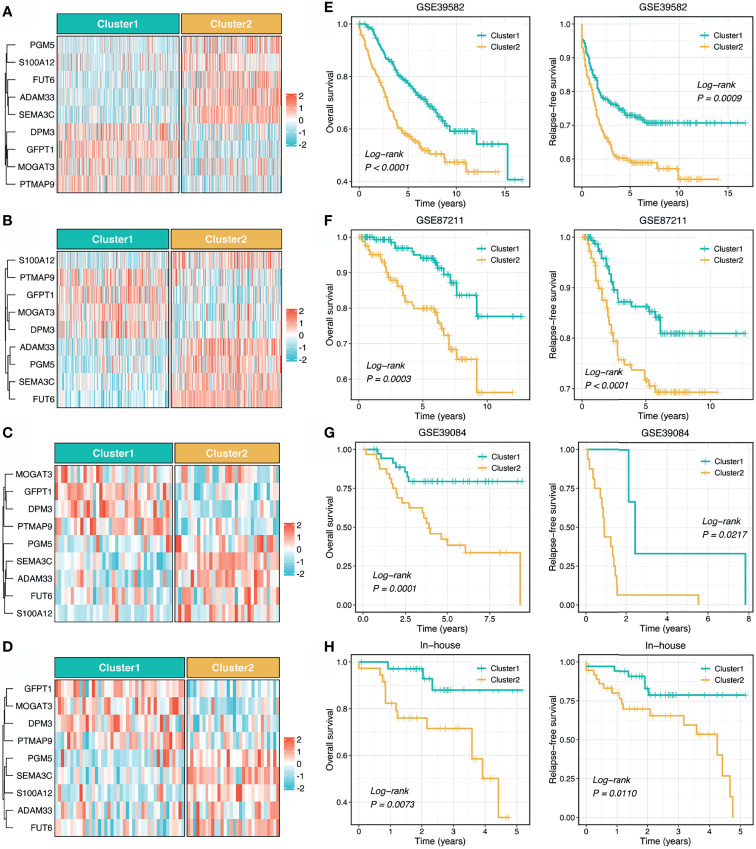
Validation and reproduction of the stemness clusters in clinical samples. **(A–D)**. Using the stemness cluster predictor, CRC patients from different cohorts were categorized into C1 or C2 in GSE39582 **(A)**, GSE87211 **(B)**, GSE39084 **(C)**, and in-house **(D)** cohorts. **(E–H)**. Kaplan-Meier curves of OS and RFS according to the stemness clusters in GSE39582 **(E)**, GSE87211 **(F)**, GSE39084 **(G)**, and in-house **(H)** cohorts.

To further explore the clinical application value of the stemness cluster predictor in clinical samples, we also enrolled three independent datasets and a clinical in-house cohort (qRT-PCR data) to verify its performance. Using the stemness cluster predictor, CRC patients from different cohorts were categorized into C1 or C2. As illustrated in **Figures 3A**–**D**, the proportions of each subtype were similar across the different cohorts. Consistent with the prior results, C2 possessed significantly dismal OS and RFS compared with C1 (**Figures 3E**–**H**), which validated the performance and reproduction of the stemness clusters in clinical samples.

### Multi-Omics Landscape of Two Stemness Clusters

**Figure 4:**
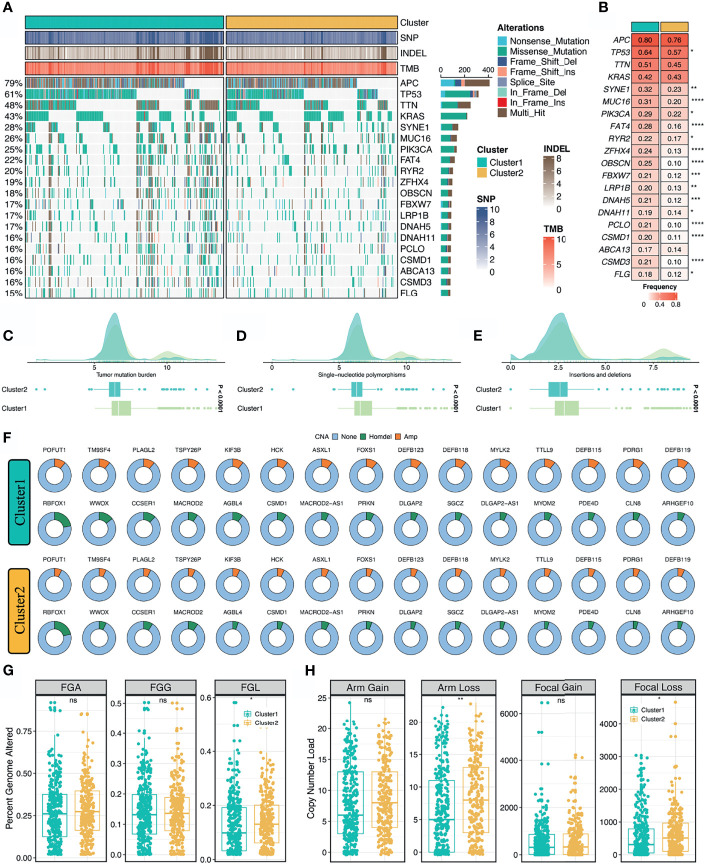
Multi-omics landscape of the stemness clusters. **(A, B)**. Mutational landscape **(A)** and frequency **(B)** of the top 20 FMGs between two clusters. **(C–E)** Distributions of TMB **(C)**, SNP **(D)**, and Indel **(E)** between two clusters. **(F)** CNV frequency of 30 FAGs/FHGs between two clusters. **(G)** Distributions of FGA, FGG, and FGL between two clusters. **(H)**. Distributions of arm gain, arm loss, focal gain, and focal loss. ^ns^ *P* > 0.05, *P < 0.05, **P < 0.01, ***P < 0.001, ****P < 0.0001.

As illustrated in **Figure 4A**, we delineated the mutational landscape of 20 FMGs in two stemness clusters. Overall, C1 exhibited significantly higher mutational frequency than C2 in most FMGs, such as *TP53*, *SYNE1*, *MUC16*, and *PIK3CA* (**Figure 4B**). Notably, *APC*, *TTN*, and *KRAS* mutations were not statistically significant between the two clusters (**Figure 4B**). In line with the mutational profiles of these FMGs, C1 also showed generally superior burden including TMB, SNPs, and Indels (*P <*0.0001, **Figures 4C**–**E**). Furthermore, we characterized the CNV status of 30 FAGs/FHGs between the two clusters. Interestingly, C1 displayed a significantly higher CNV rate for 15 FAGs, but no clear differences for 15 FHGs compared with C2 (**Figure 4F**). Subsequently, we further measured the overall genomic alteration in bases, fragments, and chromosome arms between the two clusters (**Figures 4G, H**). There were no differences in FGA, FGG, arm gain and focal gain, which suggested two clusters behaved similarly in terms of copy number amplification (**Figures 4G, H**). For another, C2 displayed a higher burden of copy number deletion at the level of bases, fragments, and chromosome arms (**Figures 4G, H**). Taken together, C1 was mutation-driven, while C2 was copy number deletion-driven.

### Underlying Biological Pathways of Two Stemness Clusters

**Figure 5:**
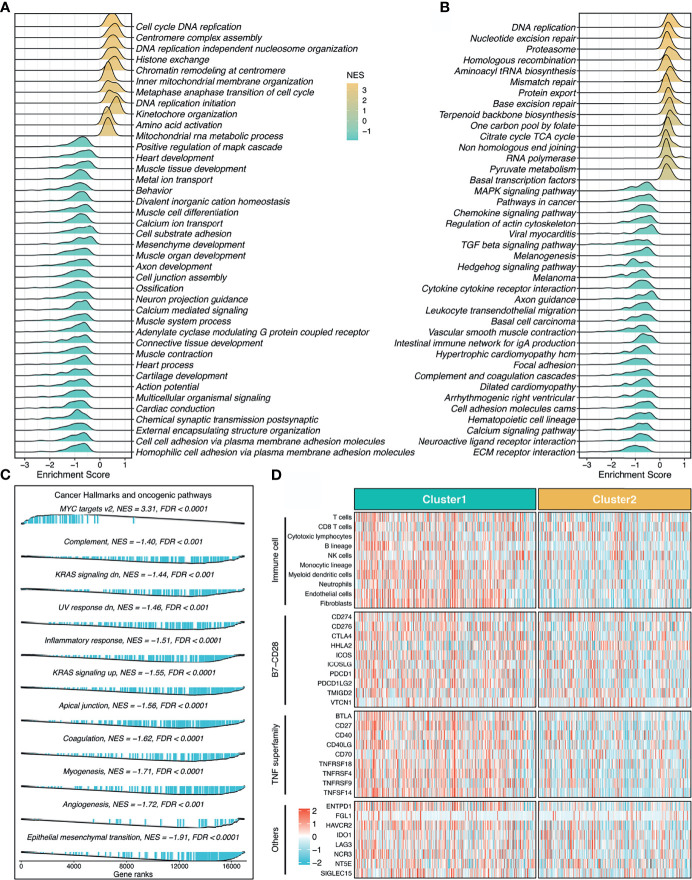
Underlying biological pathways and distinct microenvironment patterns of two stemness clusters. **(A–C)**. GO **(A)**, KEGG **(B)**, and cancer hallmark **(C)** of gene set enrichment analysis (GSEA) according to the stemness clusters. **(D)**. The immune cell infiltrations and immune checkpoint profiles of two clusters.

To explore the latent biological behavior of each stemness cluster, we performed GSEA analysis on GO annotation and KEGG pathways. GSEA-GO illustrated that C1 was characterized by proliferation-related processes (e.g., cell cycle DNA replication), while C2 significantly enriched abundant functions associated with mesenchyme development and differentiation (e.g., mesenchyme development) (**Figure 5A**). Likewise, GSEA-KEGG pathways displayed similar results in two clusters (**Figure 5B**). Additionally, further cancer hallmarks analysis revealed a proliferation-related pathway, MYC targets v2 was significantly unregulated in C1, while C2, as a subtype with high stemness characteristics, was enriched for multiple oncogenic pathways, such as epithelial mesenchymal transition, angiogenesis, myogenesis, and apical junction (**Figure 5C**).

### Distinct Microenvironment Patterns Between the Two Stemness Clusters

The MCP-counter algorithm was utilized to quantify the absolute infiltration abundance of eight immune and two stromal cell subpopulations from the transcriptomic profiles. As displayed in **Figure 5D**, C1 behaved as the immune-hot subtype, and was characterized by abundant infiltration of microenvironment cells, such as T cells, cytotoxic lymphocytes, B lineage, nature killer cells, monocytic lineage, myeloid dendritic cells, neutrophils, endothelial cells, and fibroblasts (*P <*0.05, **Figure S3**). Furthermore, C1 also had predominantly higher expression levels of immune checkpoint molecules relative to C2, such as *PD-1*, *PD-L1*, and *CTLA-4* (**Figure 5D** and **Figure S3**). The distinct microenvironment patterns between the two stemness clusters might explain their clinical outcomes and hint at the application prospect of precision immunotherapy.

### Stemness Cluster 1 Possessed Better Immunotherapeutic Efficacy

**Figure 6:**
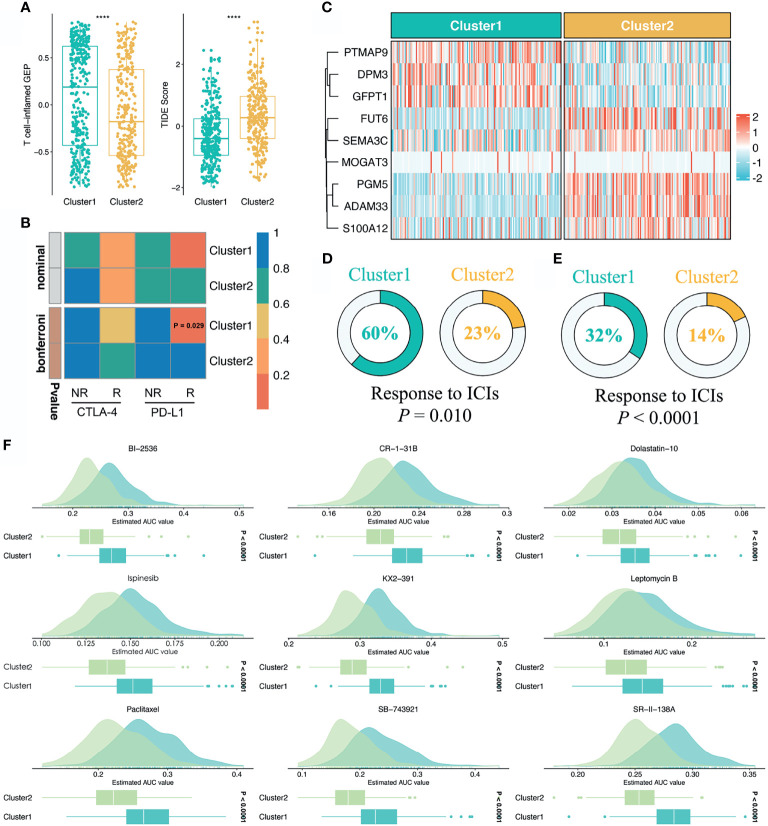
Sensitivity of two clusters to immunotherapy and chemotherapy. **(A)** Distributions of T-cell-inflamed GEP and TIDE prediction score between two clusters. ****P < 0.0001. **(B)** SubMap analysis manifested that the C1 could be more sensitive to the anti-PD-1 therapy (Bonferroni *P* = 0.029). **(C)** Using the stemness cluster predictor, CRC patients from IMvigor 210 were categorized into C1 or C2. **(D, E)** Distributions of responders and nonresponders between two clusters in GSE35640 and IMvigor 210 datasets. **(F)** Identification of nine potential therapeutic agents for C2.

To further assess the immunotherapeutic efficacy between the two stemness clusters, we applied three different algorithms to quantify the response differences. In this study, C1 presented a higher level of T-cell-inflamed GEP, suggesting activated immune status and elevated response to immune checkpoint inhibitor (ICI) treatment (*P <*0.0001, **Figure 6A**). TIDE analysis revealed C1 also had a stronger potential of tumor immune evasion and worse immunotherapeutic efficacy (*P <*0.0001, **Figure 6A**). In the SubMap framework, C1 demonstrated the similar expression patterns with the group responding to *PD-L1* inhibitors (**Figure 6B**). Subsequently, we utilized two immunotherapy cohorts with both expression data and immunotherapeutic information, to further investigate the immunotherapeutic value of two stemness clusters in clinical samples. Using our stemness cluster predictor, we divided these clinical samples into two stemness clusters (**Figure S4** and **Figure 6C**). Notably, C1 possessed a significantly superior proportion of responders than C2 in GSE35640 and IMvigor 210 datasets (*P <*0.05, **Figures 6D, E**).

### Identification of Potential Therapeutic Agents for Stemness Cluster 2

Based on the large-scale drug sensitivity and expression data from the CTRP and PRISM databases, we developed a ridge regression model *via* the *pRRophetic* package to predict the response of numerous drugs. Afterwards, this model was utilized to estimate drug sensitivity in the TCGA-CRC dataset. To identify potential therapeutic agents for C2, we performed the differential drug response analysis between the two stemness clusters. Ultimately, nine drugs with the thresholds of FDR <0.05 and log_2_FC >0.15 were considered candidate agents for C2, namely BI-2356, CR-1-31B, Dolastatin-10, Ispinesib, KX2-391, Leptomycin B, Paclitaxel, SB-743921, and SR-II-138A (**Figure 6F**). All these agents demonstrated lower estimated AUC values in C2, which suggested the great potential to develop several promising compounds for C2.

## Discussion

This study systematically established the links between the intertumoral heterogeneity and cancer stemness. Unlike previous studies (9, 11), we retrieved a total of 26 published stemness signatures derived by alternative approaches (21). GSVA was performed to estimate the enrichment abundance of each signature, which generally avoided unbalanced bias of large-scale data. Subsequently, an unsupervised clustering method, NMF, was utilized to detect heterogeneous stemness clusters in 616 bulk samples of the TCGA dataset. Eventually, two clusters were identified with the aid of multiple measurement indexes. In brief, C2 possessed significantly higher stemness abundance compared with C1, which suggested an enhanced cancer stemness trait for C2. Thus, we referred to C1 as “low stemness subtype” and C2 as “high stemness subtype”. Further prognostic and clinical analysis revealed that C2 possessed dismal prognosis and more advanced tumors, which were in line with the properties of high stemness tumors (5, 19).

The two stemness clusters presented distinct genomic alterations in multi-omics levels. Whether tumor stemness and molecular variation occur successively or whether the two are in a dynamic interactive process needs to be further demonstrated. From a global perspective, C1 displayed significantly higher of TMB, SNPs, and Indels, while C2 had superior copy number deletion at the level of bases, fragments, and chromosome arms, suggesting that C1 was mutation-driven and C2 was copy number deletion-driven. The high burden of copy number deletion was reported to be correlated with immune evasion, high proliferation trait, and worse prognosis (36). In this study, malignant phenotype C2 also showed numerous proliferation-related pathways and less immune infiltration. C1 referred to the low stemness subtype with the improved clinical outcomes, displayed predominant mutations in multiple oncogenes and tumor suppressors, such as *TP53*, *SYNE1*, *MUC16*, and *PIK3CA*. The high mutational load tends to produce more neoantigens, which could further induce the proliferation and activation of T cell to eliminate tumor cells (37). Correspondingly, C1 belonged to the “immune-hot” subtype with the abundant enrichment of various immune cells and immune checkpoint molecules. In addition, MSI-H tumors are broadly reported to be associated with stronger cytolytic activity and better immunotherapeutic response, were also enriched in C1. Based on the CMS information inferred from the *CMSclassifier* package, we observed that a higher proportion of CMS1 in C1 and of CMS4 in C2. As is well known, CMS1 and CMS4 belong to the microsatellite instability immune subtype and mesenchymal subtype, respectively (35). Taken together, these results indicated that C1 had a great potential to benefit from immunotherapy relative to C2.

Subsequently, three different algorithms, including T-cell-inflamed GEP, TIDE, and SubMap analysis, were performed to evaluate assess the immunotherapeutic efficacy between the two stemness clusters. Consistently, C1 was more prone to generate efficacy from immunotherapy. To further investigate the immunotherapeutic value of two stemness clusters in clinical samples, we enrolled two immunotherapy cohorts with both expression data and immunotherapeutic information. As previously described, C1 possessed a significantly superior proportion of responders than C2 in two cohorts. Thus, our stemness clusters provided novel insight into precise immunotherapy.

As mentioned above, C2 belongs to the high stemness subtype with worse prognosis, thus, more treatments are needed to improve its clinical outcomes. To bridge this gap, we developed a ridge regression model using the large-scale drug sensitivity and expression data. Ultimately, this model identified nine potential therapeutic agents for C2, including BI-2356, CR-1-31B, Dolastatin-10, Ispinesib, KX2-391, Leptomycin B, Paclitaxel, SB-743921, and SR-II-138A. All these agents demonstrated significantly higher sensitivity in C2, which suggested the great potential to develop several promising compounds for C2. Dolastatin-10, a pentapeptide isolated from the marine mollusk Dolabella Auricularia with latent antitumor properties, could effectively induce tumor apoptosis (38). Bousquet et al. have reported that Dolastatin-10 could inhibit oncogenic *KRAS* and hypoxia-inducible factors pathways in CRC (39). Leptomycin B serves as a potent and specific inhibitor of nuclear export that promotes G1 cell cycle arrest in tumor cells (40). Paclitaxel, a natural flavonoid, interacts with cell cycle modulators and leads to cell cycle arrest by activating the Wnt/β-catenin signaling pathway (41). Although CRC cells are prone to be resistant to Paclitaxel (42), C2 could be a sensitive subtype. Given the above, these drugs or combination therapies could enhance the treatment efficiency, which brought more effective treatment strategies to improve the clinical outcomes of C2. Current works shed new light on delivering precision medicine for CRC.

To the best of our knowledge, this is the first and most comprehensive study to date identifying the heterogeneous stemness clusters according to the large-scale data. To reproduce our subtype in other cohorts, we developed a stemness cluster predictor consisting of nine genes *via* four machine learning algorithms. The stemness cluster predictor remained excellent performance in the training and testing sets. To further translate the stemness cluster predictor into clinical settings, three independent datasets and a clinical in-house cohort (qRT-PCR data) were utilized to verify its performance. Using the stemness cluster predictor, the proportions of each subtype were similar across the different cohorts. In parallel, C2 possessed significantly dismal OS and RFS compared with C1, which validated and reproduced the stemness clusters in clinical samples. Although the stemness cluster was promising, some limitations should be acknowledged. First, all samples enrolled in this study were retrospective, and prospective studies are further needed to confirm our conclusions. Second, the validation dataset lacked multi-omics data to explore differences in genomic alterations between the two stemness clusters in different populations. Third, the dynamic interactive process between the stemness clusters and multi-omics alterations needs to be further explored. Fourth, the immunotherapeutic efficacy was evaluated by bioinformatics algorithms and non-CRC clinical samples, which might not accurately reflect the difference in immunotherapy response between the two stemness clusters, and further investigation should be conducted.

In conclusion, this study established the links between the intertumoral heterogeneity and cancer stemness in CRC. We proposed two stemness clusters with distinct clinical outcomes, multi-omics landscape, biological mechanisms, and immune features of the stemness clusters. C1 was more sensitive to immunotherapy relative to C2. For patients with C2, our study provided latent therapeutic drugs to them, which might improve their clinical outcomes. Overall, this study has not only provided new insights into the heterogeneity of cancer stemness, but also thrown light on optimizing decision-making in immunotherapy and chemotherapy for CRC patients.

## Data Availability Statement

Public data used in this work can be acquired from the TCGA portal (https://portal.gdc.cancer.gov/) and GEO (http://www.ncbi.nlm.nih.gov/geo/) under the accession numbers GSE39582, GSE87211, GSE39084 and GSE35640. The raw experimental data supporting the conclusions of this article will be made available by the corresponding author.

## Ethics Statement

The human cancer tissues used in this study were approved by Ethnics Committee of The First Affiliated Hospital of Zhengzhou University. The participants provided their written informed consent to participate in this study.

## Author Contributions

ZL and XH designed this work. ZL integrated and analyzed the data. ZL wrote this manuscript. ZL, HX, SW, YR, and XH edited and revised the manuscript. All authors contributed to the article and approved the submitted version.

## Funding

This study was supported by the Key Research Projects of Henan Higher Education (No.16A320053) and the Youth Innovation Fund of The First Affiliated Hospital of Zhengzhou University.

## Conflict of Interest

The authors declare that the research was conducted in the absence of any commercial or financial relationships that could be construed as a potential conflict of interest.

## Publisher’s Note

All claims expressed in this article are solely those of the authors and do not necessarily represent those of their affiliated organizations, or those of the publisher, the editors and the reviewers. Any product that may be evaluated in this article, or claim that may be made by its manufacturer, is not guaranteed or endorsed by the publisher.
