# Supplementary material for: Stemness Refines the Classification of Colorectal Cancer With Stratified Prognosis, Multi-Omics Landscape, Potential Mechanisms, and Treatment Options
Source: Front Immunol. 2022 Jan 27;13:828330. doi: 10.3389/fimmu.2022.828330 (PMC8828967; doi:10.3389/fimmu.2022.828330)
Supplement: Supplementary file 1 [file DataSheet_1.docx]

**Supplementary material**

- Figure S1
- Figure S2
- Figure S3
- Figure S4
- Supplementary method

**
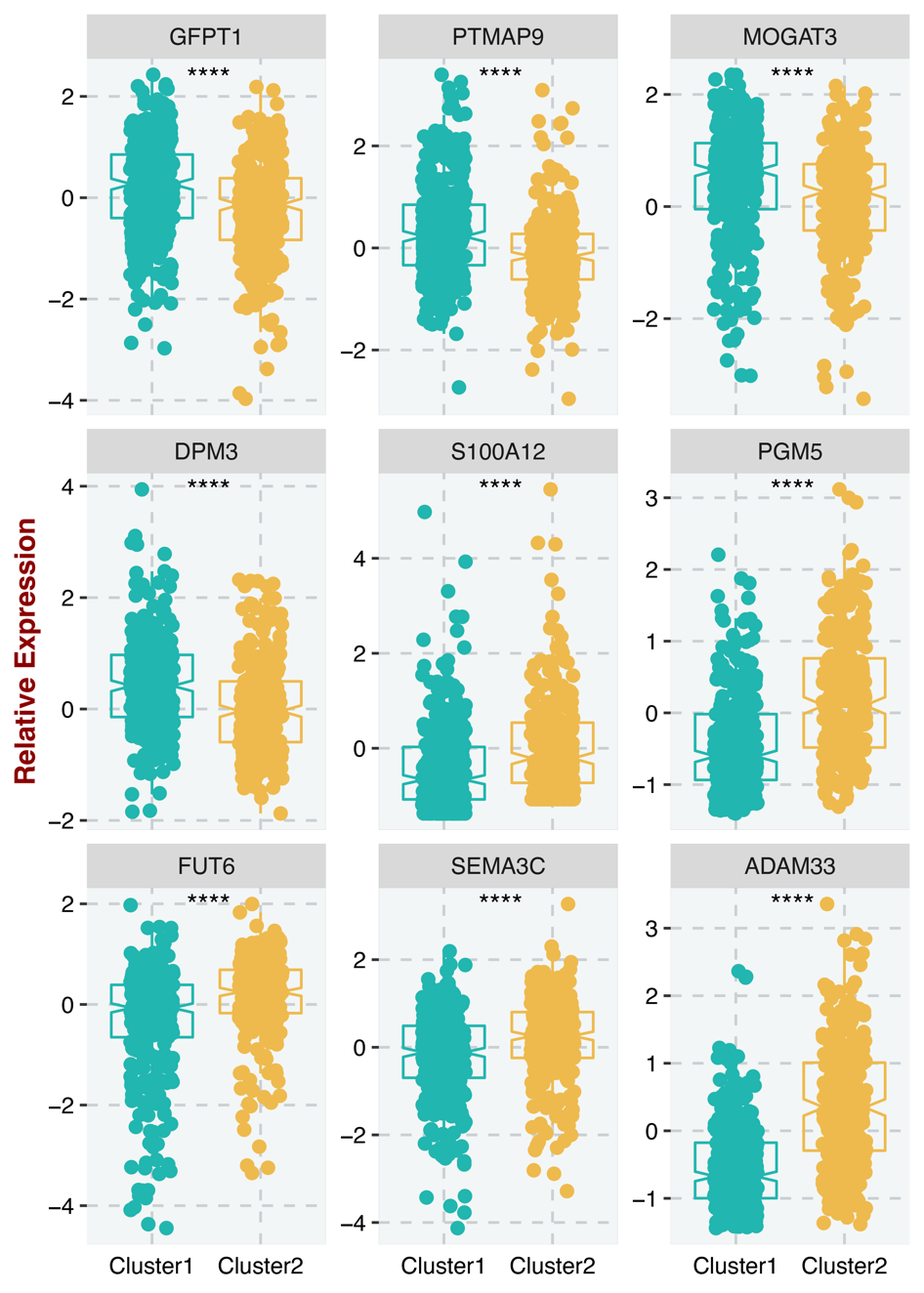
**

**Figure S1.** Distribution of nine key stemness predictor genes between two stemness clusters. *****P* <0.0001.

**
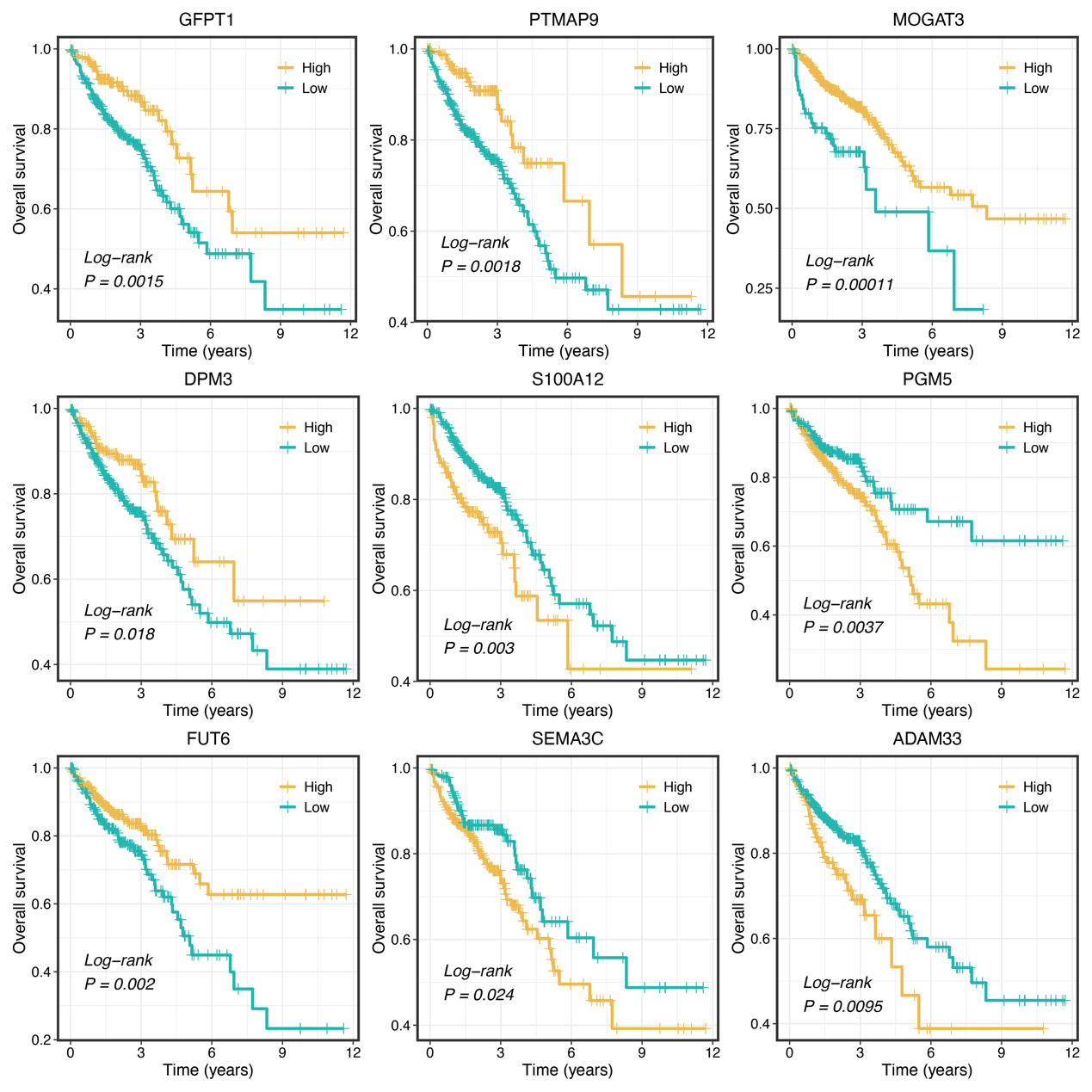
**

**Figure S2.** Kaplan-Meier curves of OS according to the nine key stemness predictor genes.

**
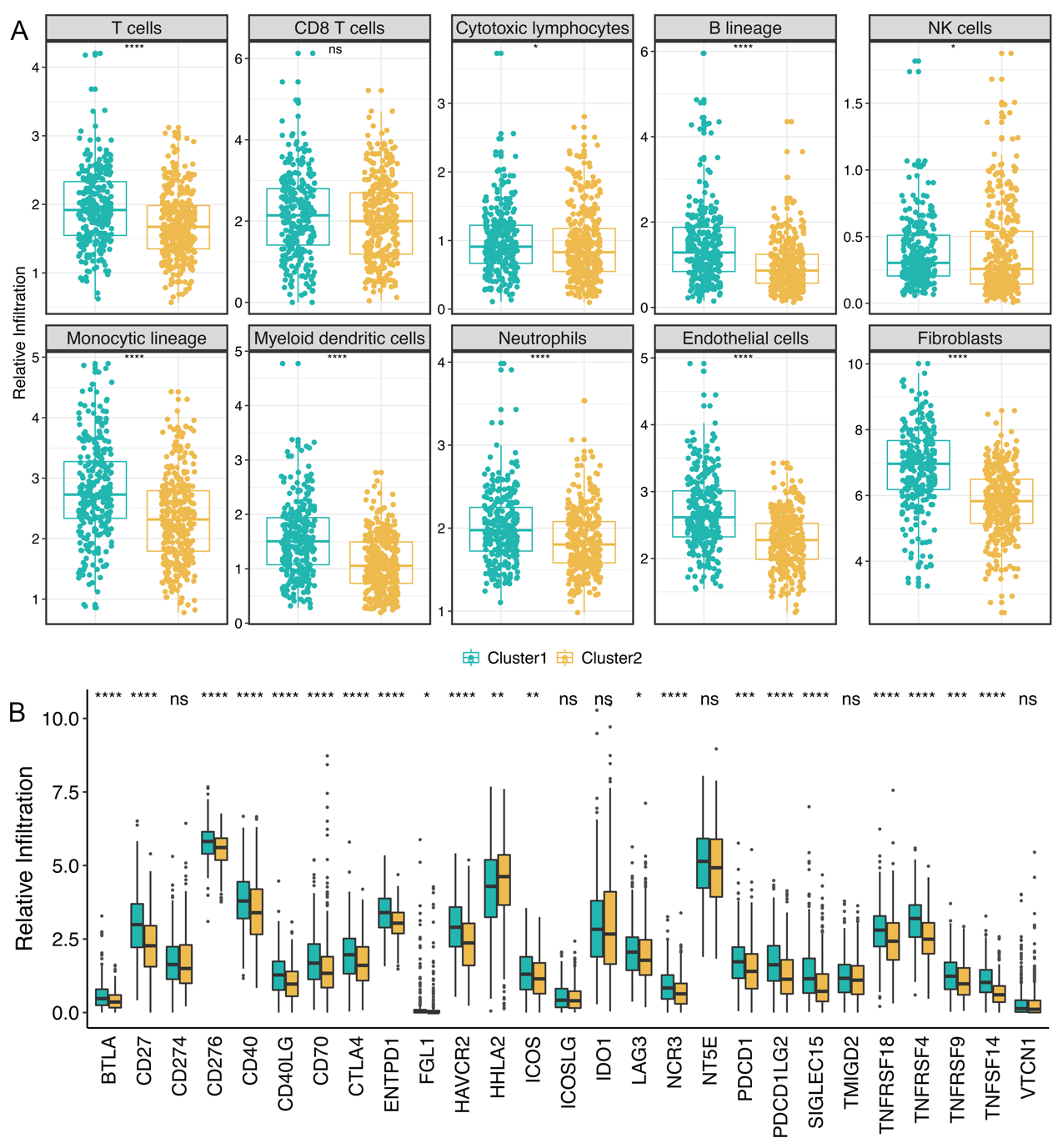
**

**Figure S3.** **Distinct microenvironment patterns between two stemness clusters. A**. Distributions of 12 immune and stromal cells between two clusters. **B**. Distributions of 27 immune checkpoint molecules between two clusters. ^ns^*P* >0.05, **P* <0.05, ***P* <0.01, ****P* <0.001, *****P* <0.0001.

**
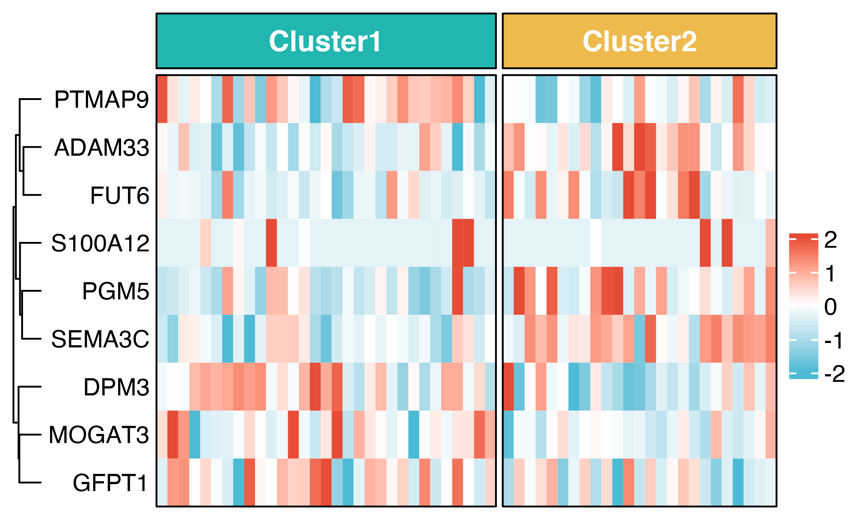
**

**Figure S4**. Using the stemness cluster predictor, CRC patients from GSE35640 were categorized into C1 or C2.

**Supplementary method**

**Quantitative Real-Time PCR (qRT-PCR)**

Total RNA was isolated from CRC tissues using RNAiso Plus reagent RNA quality was evaluated using a NanoDrop One C (Waltham, MA, USA), and RNA integrity was assessed using agarose gel electrophoresis. An aliquot of 1 μg of total RNA was reverse transcribed into complementary DNA (cDNA) according to the manufacturer's protocol using a High-Capacity cDNA Reverse Transcription kit (TaKaRa BIO, Japan). qRT-PCR was performed using SYBR Assay I Low ROX (Eurogentec, USA) and SYBR® Green PCR Master Mix (Yeason, Shanghai, China) to detect the expression of 16 lncRNAs expression. The expression value was normalized to *GAPDH*, and then log2 transformed for subsequent analysis. The primer sequences of the included nine genes and *GAPDH* were shown in Table S2.
